# Cytogenetics and gene mutations influence survival in older patients with acute myeloid leukemia treated with azacitidine or conventional care

**DOI:** 10.1038/s41375-018-0257-z

**Published:** 2018-10-01

**Authors:** Hartmut Döhner, Anna Dolnik, Lin Tang, John F. Seymour, Mark D. Minden, Richard M. Stone, Teresa Bernal del Castillo, Haifa Kathrin Al-Ali, Valeria Santini, Paresh Vyas, C. L. Beach, Kyle J. MacBeth, Barry S. Skikne, Steve Songer, Nora Tu, Lars Bullinger, Hervé Dombret

**Affiliations:** 1 Ulm University Hospital, Ulm, Germany; 2 Celgene Corporation, Summit, NJ United States; 3 Peter MacCallum Cancer Centre, Royal Melbourne Hospital, Melbourne, and University of Melbourne, Parkville, Australia; 4 University of Toronto, Toronto, ON Canada; 5 Dana-Farber Cancer Institute, Boston, MA United States; 6 Hospital Central de Asturias, Oviedo, Spain; 7 Universitätsklinikum Halle (Saale), Halle, Germany; 8 AOU Careggi, University of Florence, Florence, Italy; 9 University of Oxford, Oxford, United Kingdom; 10 Charité University Medicine, Berlin, Germany; 11 Hôpital Saint Louis, Institut Universitaire d’Hématologie, Université Paris Diderot, Paris, France

## Abstract

Older patients with newly diagnosed acute myeloid leukemia (AML) in the phase 3 AZA-AML-001 study were evaluated at entry for cytogenetic abnormalities, and a subgroup of patients was assessed for gene mutations. Patients received azacitidine 75 mg/m^2^/day x7 days (*n* = 240) or conventional care regimens (CCR; *n* = 245): intensive chemotherapy, low-dose cytarabine, or best supportive care only. Overall survival (OS) was assessed for patients with common (occurring in ≥10% of patients) cytogenetic abnormalities and karyotypes, and for patients with recurring gene mutations. There was a significant OS improvement with azacitidine vs CCR for patients with European LeukemiaNet-defined Adverse karyotype (HR 0.71 [95%CI 0.51–0.99]; *P* = 0.046). Azacitidine-treated patients with -5/5q-, -7/7q-, or 17p abnormalities, or with monosomal or complex karyotypes, had a 31–46% reduced risk of death vs CCR. The most frequent gene mutations were *DNMT3A* (27%), *TET2* (25%), *IDH2* (23% [R140, 15%; R172, 8%]), and *TP53* (21%). Compared with wild-type, OS was significantly reduced among CCR-treated patients with *TP53* or *NRAS* mutations and azacitidine-treated patients with *FLT3* or *TET2* mutations. Azacitidine may be a preferred treatment for older patients with AML with Adverse-risk cytogenetics, particularly those with chromosome 5, 7, and/or 17 abnormalities and complex or monosomal karyotypes. The influence of gene mutations in azacitidine-treated patients warrants further study.

## Introduction

Acute myeloid leukemia (AML) is associated with a range of recurring cytogenetic abnormalities and gene mutations [1–4]. While the prognostic importance of cytogenetics in AML has been established for decades [5], due to recent advances in next-generation sequencing and greater availability of myeloid-focused gene panels, some genes frequently mutated in AML have been identified that are predictive of treatment response [2, 3, 6]. Molecular genetic data are increasingly being used to inform disease classification, risk stratification, and clinical care of patients [4, 7]. Two provisional entities, AML with mutated *RUNX1* and AML with *BCR-ABL1*, have been included in the 2016 update of the World Health Organization (WHO) classification of myeloid neoplasms and acute leukemia [7]. Mutational testing for *NPM1*, *CEBPA*, and *FLT3* is advised in the 2010 European LeukemiaNet (ELN) recommendations for AML [1], and the 2017 update to the ELN recommendations lists three additional genes—*RUNX1*, *ASXL1*, and *TP53*—that can inform risk stratification, mainly based on experience with intensive chemotherapy (IC) in relatively younger patients [4]. Patterns of co-mutations have also been identified that have distinct prognostic implications in AML [3].

In the randomized, phase 3 AZA-AML-001 study of older patients with newly diagnosed AML (NCT01074047), azacitidine prolonged median overall survival (OS) vs conventional care regimens (CCR) (10.4 vs 6.5 months, respectively; *P* = 0.101), with 1-year survival rates of 46.5% vs 34.2%, respectively [8]. A prospective sub-analysis from the study showed a significantly prolonged OS of 3.2 months with azacitidine compared with CCR (hazard ratio [HR] 0.68, 95% confidence interval [95%CI] 0.50, 0.94) in the subgroup of patients with poor-risk cytogenetics, as defined by National Comprehensive Cancer Network (NCCN) 2009 criteria [8, 9]. That analysis did not investigate outcomes associated with specific cytogenetic abnormalities.

Here we evaluate survival outcomes in patient subgroups from the AZA-AML-001 study, defined by 2010 ELN cytogenetic risk classification and by the presence of specific cytogenetic abnormalities or gene mutations at baseline. Pretreatment cytogenetic risk classification was an entry criterion and cytogenetic data were available for almost all patients. A subpopulation of patients in the AZA-AML-001 study with available baseline bone marrow samples for molecular analyses consented to participate in exploratory analyses, to evaluate the frequency of recurring gene mutations at entry and relationships between pretreatment mutational status and OS.

## Methods

### Study design

Full study design, patient eligibility criteria, and response endpoints are described in detail elsewhere [8]. Briefly, patients aged ≥65 years with newly diagnosed AML, >30% bone marrow blasts, Eastern Cooperative Oncology Group performance status (ECOG PS) scores ≤2, white blood cell counts ≤15 × 10^9^/L, and intermediate- or poor-risk cytogenetics per 2009 NCCN guidelines for AML [9], were eligible to participate. This study was approved by all relevant institutional review boards or independent ethics committees and was conducted according to the Declaration of Helsinki. All patients provided written informed consent.

Patients were preselected to 1 of 3 CCR: IC (cytarabine 100–200 mg/m^2^ IV for 7 days + anthracycline IV for 3 days induction), low-dose cytarabine (LDAC; 20 mg SC BID for 10 days per 28-day cycle), or best supportive care (BSC) only. After preselection, patients were randomized 1:1 to azacitidine (75 mg/m^2^/day SC for 7 consecutive days per 28-day cycle) or to CCR; those randomized to CCR received their preselected regimen. All patients could receive BSC as needed.

### Cytogenetic analyses

**Fig. 1:**
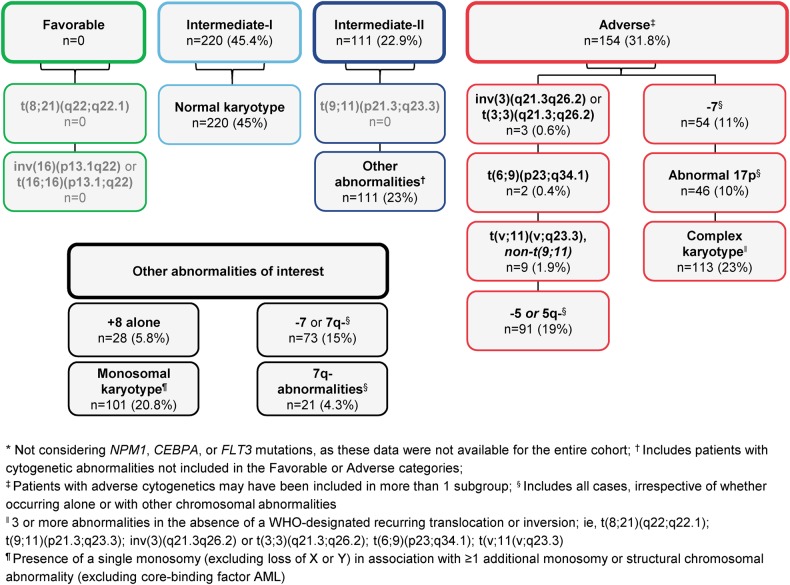
Patient subgroups according to modified 2010 ELN criteria* and frequency of specific chromosomal abnormalities or karyotypes

Karyotypes from pretreatment bone marrow samples were determined locally and karyograms were prepared and sent for central review by an independent cytogeneticist (Anne Hagemeijer, MD). For these analyses, cytogenetic risk status was determined according to modified 2010 ELN recommendations [1], but molecular markers were not considered for risk-group assessments, as they were not available for all patients. Patient subgroups were identified based on ELN-defined cytogenetic risk classifications: Intermediate-I (normal karyotype), Intermediate-II (comprising all abnormalities not classified as Favorable or Adverse), and Adverse (Fig 1). OS outcomes associated with specific cytogenetic abnormalities observed in ≥10% of patients, and with complex or monosomal karyotypes, were also evaluated. Complex karyotype was defined as three or more cytogenetic abnormalities in the absence of a WHO-designated recurring translocation or inversion; i.e., t(8;21)(q22;q22.1); t(9;11)(p21.3;q23.3); inv(3)(q21.3q26.2) or t(3;3)(q21.3;q26.2); t(6;9)(p23;q34.1); t(v;11;q23.3). Monosomal karyotype was defined as the presence of a single monosomy (excluding loss of one X or Y chromosome) in association with one or more additional monosomy or structural chromosomal abnormality. Patients with multiple lesions may have been assigned to and evaluated in more than one category. Because the majority (64%) of all patients in AZA-AML-001 were preselected to receive LDAC before randomization, OS was also compared among LDAC-preselected patients who received azacitidine vs those who received LDAC. The small number of patients preselected to BSC or IC precluded statistical comparisons between cytogenetic abnormality subgroups.

### Mutational analyses

DNA was isolated from pretreatment bone marrow mononuclear cells and targeted sequencing of 39 genes was performed with Haloplex target enrichment (Agilent) on Illumina HiSeq 2500 using 2 × 100 bp read lengths. Burrows-Wheeler Aligner (BWA)-MEM alignment to genome (hg19) and VarScan v2.3.9 software (Genome Institute at Washington University), a platform-independent tool, were used to detect variants. Target regions varied by gene from all exons to hotspots. Variant annotation filtering included functionally deleterious variants (SnpEff v4.0) functional prediction as non-synonymous SNV/INDEL in exons, splicing regions, and stop sites. Pindel (v0.2.5b5) was used to detect deletions, inversions, small insertions, and tandem duplicates with the parameter setting at a minimum coverage of 10 with a minimum of 5 supporting reads. Heterozygous variant allele frequency (VAF) detection threshold was 3%. There was a total of 312 variants in the combined Pindel and VarScan results. *FLT3* tyrosine kinase domain (TKD) mutations were determined by next-generation sequencing, and internal tandem duplications (ITD) were determined by capillary electrophoresis sizing of polymerase chain reaction (PCR) amplicons from exons 14 and 15 (binary call; no allelic ratio data were available).

### Statistical methods

Median OS and 1-year survival rates are estimated using Kaplan–Meier methods. OS comparisons according to 2010 ELN cytogenetic risk status, specific cytogenetic abnormalities, and karyotype are made using a weighted log-rank test. HRs and 95%CIs are from an unstratified Cox proportional hazards model; *P* values are from weighted log-rank tests (not adjusted for multiplicity of testing).

Within each treatment arm (azacitidine or CCR), OS was compared between patients with specific gene mutations vs those with corresponding wild-type genes. Additionally, OS comparisons were made between the azacitidine and CCR arms for patients with gene mutations detected in ≥5 patients. OS comparisons by gene mutational status are made using a log-rank test stratified by baseline ECOG PS score (0–1 vs 2) and NCCN cytogenetic risk (intermediate vs poor).

The influence of VAFs of gene mutations found to significantly influence OS in univariate analyses was investigated in two Cox proportional hazards models [10]. Relative hazards were simulated for VAF from the Cox proportional hazards models and plotted against the VAF to visualize the effect of VAF on OS. One model evaluated relative OS hazards by mutant VAF as a continuous variable vs OS in patients with wild-type corresponding genes (VAF = 0), with treatment (azacitidine, CCR) as strata in the model. The second model investigated the relative OS hazards by baseline mutation VAFs vs wild-type genes within the azacitidine and CCR arms. When multiple loci were mutated within a gene, the mutation with the highest VAF was used in the model.

## Results

### Patients

The intention-to-treat population in AZA-AML-001 included 488 patients (azacitidine, *n* = 241; CCR, *n* = 247) [8]. Of them, centrally reviewed cytogenetic data were available for 485 patients (99.4%; azacitidine, *n* = 240; CCR, *n* = 245, including IC [*n* = 44], LDAC [*n* = 158], and BSC only [*n* = 45]). In all, 220 patients (45.4%) had ELN-defined Intermediate-I risk (i.e., normal) karyotype (azacitidine, *n* = 114; CCR, *n* = 106), 111 patients (22.9%) had an Intermediate-II risk karyotype (azacitidine, *n* = 53; CCR, *n* = 58), and 154 patients (31.8%) had an Adverse risk karyotype (azacitidine, *n* = 73; CCR, *n* = 81) (Fig. 1). Baseline characteristics of the cytogenetic analysis population were essentially unchanged from those of all patients in the AZA-AML-001 study [8].

The “biomarker cohort” comprised 156 patients who were assessed at study entry for presence of gene mutations (azacitidine, *n* = 83; CCR, *n* = 73). Baseline characteristics were generally similar between azacitidine-treated and CCR-treated patients (Supplementary Table 1). Prior history of myelodysplastic syndromes (MDS) was somewhat more common in azacitidine-treated patients (23% vs 14% of CCR patients) and CCR-treated patients were proportionally more likely to have ELN-defined Adverse risk cytogenetics (44% vs 33%).

### Cytogenetic analyses

**Fig. 2:**
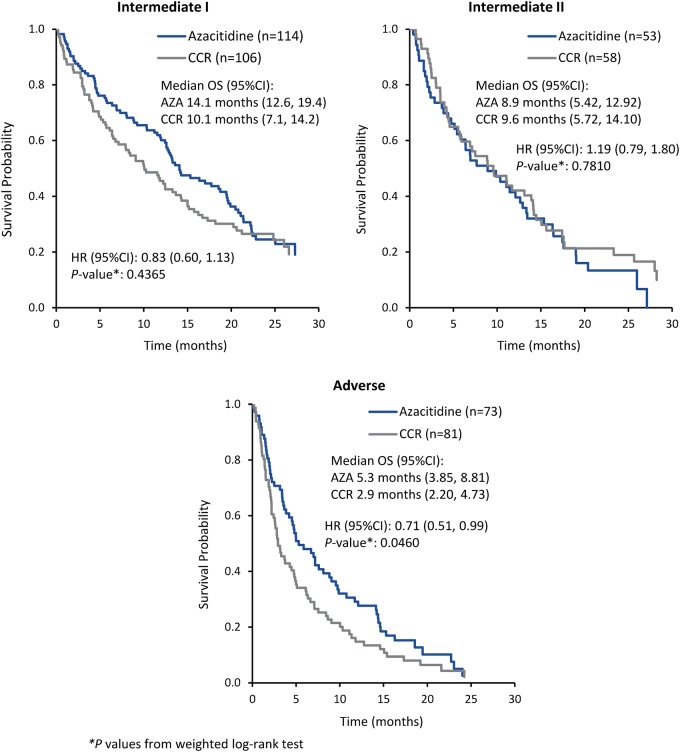
Overall survival associated with cytogenetic risk groups (per modified 2010 ELN criteria)

Median OS was comparable between azacitidine and CCR among patients with Intermediate-I risk (14.1 vs 10.1 months, respectively; HR 0.83 [95%CI 0.60, 1.1]; *P* = 0.44) or Intermediate-II risk (8.9 vs 9.6 months; HR 1.19 [95%CI 0.79, 1.8]; *P* = 0.78) cytogenetics (Fig. 2). Estimated 1-year survival rates in the Intermediate-I risk group were 60.1% with azacitidine and 45.5% with CCR, and in the Intermediate-II group were 41.5% and 42.1%, respectively. There was a significant difference in median OS in favor of azacitidine among patients with Adverse risk karyotypes (5.3 vs 2.9 months with CCR; HR 0.71 [95%CI 0.51, 0.99]; *P* = 0.046), with estimated 1-year survival rates of 29.1% vs 14.7% for patients treated with azacitidine and CCR, respectively.

The LDAC preselection group included 154 patients treated with azacitidine and 158 patients treated with LDAC. Median OS with azacitidine and LDAC was 13.3 vs 12.5 months, respectively (HR 1.1 [95%CI 0.75, 1.6]), among patients with Intermediate-I risk cytogenetics, 10.7 vs 5.6 months (0.93 [0.56, 1.5]) among patients with Intermediate-II risk cytogenetics, and 5.9 vs 4.3 months (0.73 [0.47, 1.1]) for those with Adverse risk karyotypes.

**Fig. 3:**
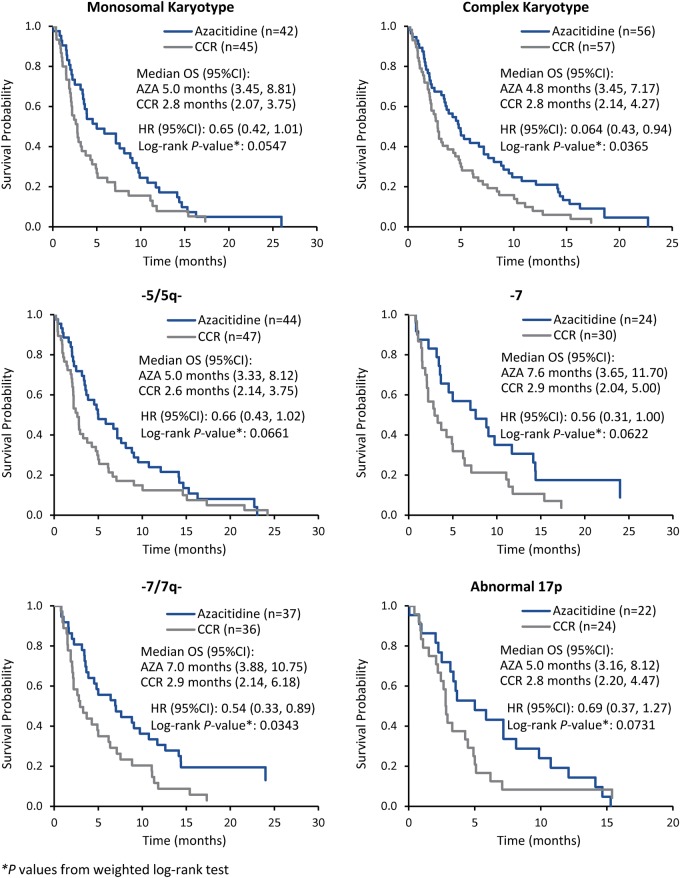
Overall survival associated with monosomal and complex karyotypes and with specific cytogenetic abnormalities occurring in ≥10% of patients

Among all patients, those with complex karyotypes (*n* = 113; 23%), those treated with azacitidine had a statistically significant improvement in OS compared with those who received CCR (median 4.8 months vs 2.8 months, respectively; HR 0.64 [95%CI 0.43, 0.94]; *P* = 0.037) (Fig. 3), with an estimated 15% more azacitidine-treated patients alive at 1 year (22.8% vs 7.9%). There was also a trend for improvement in median OS with azacitidine for patients with monosomal karyotypes (*n* = 101, 21%) (5.0 vs 2.8 months with CCR; HR 0.65 [95%CI 0.42, 1.01]; *P* = 0.055), with estimated 1-year survival rates of 19.6% vs 7.8%, respectively. Within the LDAC preselection group, median OS among patients with complex karyotypes was 5.3 months with azacitidine vs 2.9 months with LDAC (HR 0.61 [95%CI 0.36, 1.0]), and for patients with monosomal karyotypes was 5.9 months vs 2.9 months, respectively (HR 0.66 [0.37, 1.2]).

Specific cytogenetic abnormalities observed in ≥10% of patients occurred in chromosomes 5 (19%), 7 (15%), and 17p (10%) (Fig. 1). Compared with CCR, treatment with azacitidine was associated with significantly longer median OS for patients with complex karyotypes or -7/7q- abnormalities, and showed a trend for improved survival in patients with monosomal karyotypes or abnormalities in chromosomes 5 and 17 (Fig. 3). Median OS in the CCR arm was less than 3 months for patients in each of these subgroups.

### Mutational analyses

**Fig. 4:**
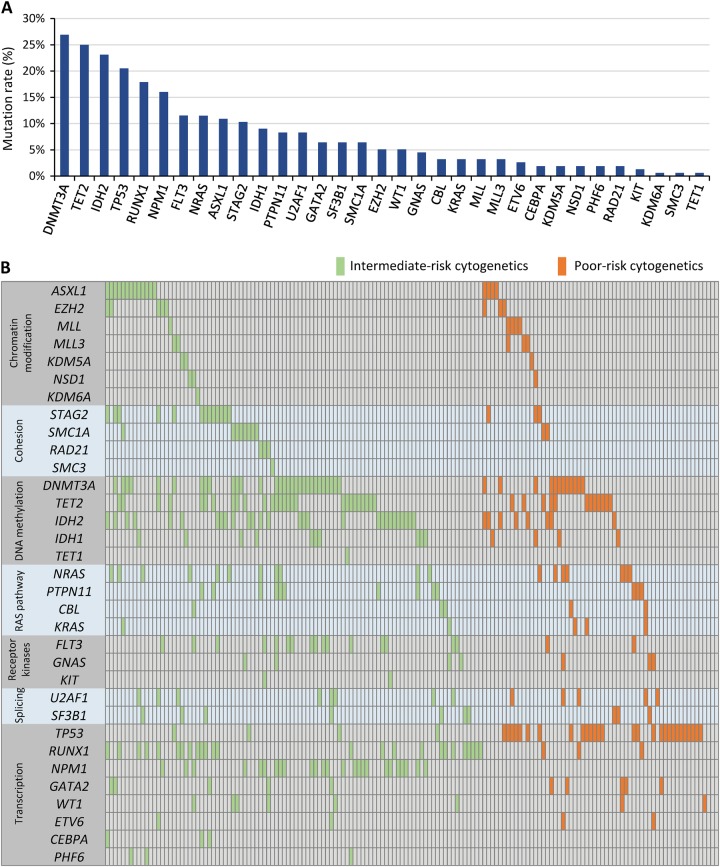
**a** Proportions of patients with specific gene mutations. **b** Oncoplot showing gene mutations in individual patients with intermediate-I/II risk (green) or poor-risk (orange) cytogenetics

Molecular abnormalities were detected in 33 of the 39 sequenced genes (Fig. 4) and in 153 (98.1%) of the 156 patients in the biomarker population. The most frequently mutated genes were *DNMT3A* (27%), *TET2* (25%), *IDH2* (23% [-R140, 15%; -R172, 8%]), *TP53* (21%), *RUNX1* (18%), *NPM1* (16%), *NRAS* (12%), *FLT3* (12% [-ITD, 10%; -TKD, 5%]), *ASXL1* (11%), and *STAG2* (10%). No mutations were found in *BRAF*, *DNMT1*, *DNMT3B*, *FAM5C*, *HNRNPK*, or *PTEN* genes. No patient with a *TP53* mutation had a co-occurring *NPM1* or *RUNX1* mutation (*P* = 0.002 and *P* = 0.001, respectively), and 50% of patients with an *FLT3* mutation also had an *NPM1* mutation (*P* < 0.001) (Supplementary Figure 1). No patient with an *NPM1* mutation had a co-occurring *RUNX1* or *ASXL1* mutation (*P* = 0.004 and *P* = 0.043, respectively). Only 3 patients had a *CEBPA* mutation and all were monoallelic.

**Fig. 5:**
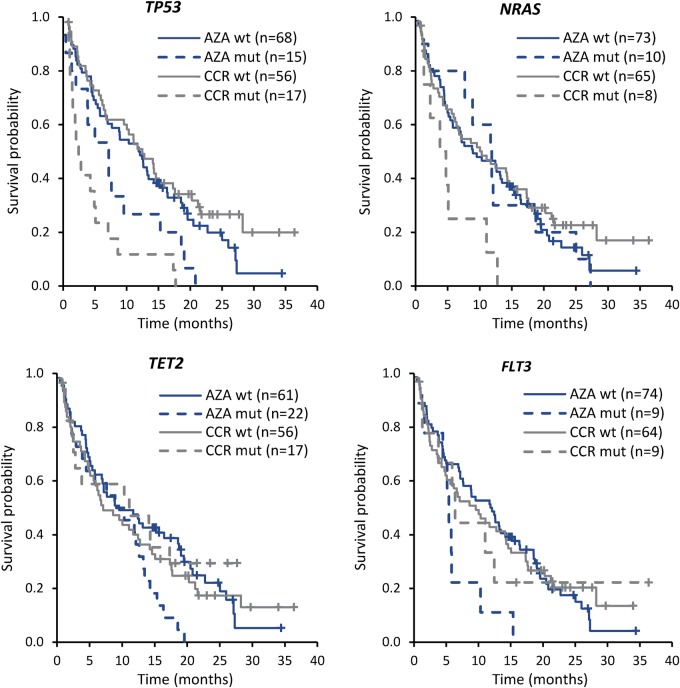
Kaplan–Meier curves for gene mutations significantly (*P* *<* 0.05) associated with overall survival within treatment arms (mutant vs wild-type)

Median OS did not differ significantly in the azacitidine and CCR arms among patients with 1, 2, or ≥3 gene mutations at study entry. Within treatment arms, four gene mutations were significantly correlated with OS compared with wild-type genes: *TP53, NRAS*, *FLT3* (including both -ITD and -TKD), and *TET2* (Table 1). In the CCR arm, median OS was significantly reduced for patients with *TP53* mutations (*n* = 17) compared with wild-type (*n* = 56) (2.4 vs 12.5 months, respectively; *P* = 0.026) and for patients with mutant *NRAS* (*n* = 8) vs wild-type *NRAS* (*n* = 65) (4.3 vs 10.3 months, respectively; *P* = 0.020) (Fig. 5). Within the azacitidine arm, median OS was not significantly different between patients with (*n* = 15) or without (*n* = 68) *TP53* mutations (7.2 vs 12.0 months, respectively; *P* = 0.40) or between patients with mutant (*n* = 10) or wild-type (*n* = 73) *NRAS* (11.8 vs 8.9 months; *P* = 0.95). However, median OS in the azacitidine arm was reduced in patients with mutant *FLT3* (*n* = 9) vs wild-type *FLT3* (*n* = 74) (5.4 vs 12.0 months, respectively; *P* = 0.017). Despite similar median OS, there was a statistically significant difference (*P* = 0.005) in OS within the azacitidine arm for patients with *TET2* mutations (*n* = 22) vs those with wild-type *TET2* (*n* = 61) due to separation of the survival curves after the median (Fig. 5). Median OS within the azacitidine and CCR treatment arms was comparable for patients with or without mutations in any of the genes known to influence DNA methylation (i.e., *DNMT3A*, *IDH1*, *IDH2*, *TET1*, and *TET2*). There were no statistically significant survival differences within either treatment arm for patients with known or provisional class-defining lesions (e.g., *RUNX1*, *NPM1*) (Table 1), or any other gene mutation evaluated compared with OS in patients with corresponding wild-type genes (Supplementary Figure 2).

**Table 1:** Median OS within treatment arms (mutant vs wild type) for the most frequently (≥10% of patients) mutated genes and genes involved in DNA methylation

Mutated gene^a^		AZA			CCR	
	WT Median OS, months (95%CI)	MUT Median OS, months (95%CI)	Stratified *P* value	WT Median OS, months (95%CI)	MUT Median OS, months (95%CI)	Stratified *P* value
*TP53*	12.0 (7.0, 16.3)	7.2 (3.9, 18.6)	0.404	12.5 (9.6, 17.6)	2.4 (1.5, 7.1)	0.026
*NRAS*	8.9 (5.8, 14.3)	11.8 (7.7, NR)	0.946	10.3 (6.4, 15.1)	4.3 (2.3, NR)	0.020
*FLT3* ^a^	12.0 (7.6, 16.3)	5.4 (4.5, NR)	0.017	9.6 (5.1, 14.6)	6.4 (3.8, NR)	0.272
*TET2*	9.5 (6.9, 18.7)	9.6 (4.5, 13.5)	0.005	7.1 (5.6, 14.2)	11.1 (2.8, NR)	0.445
*IDH2*	9.2 (7.0, 13.3)	12.6 (4.4, NR)	0.602	6.8 (4.9, 14.1)	12.5 (5.6, NR)	0.466
*DNMT3A*	8.2 (4.8, 14.3)	12.6 (7.0, 20.8)	0.413	8.6 (5.1, 14.3)	10.3 (3.8, NR)	0.597
*RUNX1*	8.3 (5.1, 13.3)	13.5 (8.8, NR)	0.718	6.1 (3.8, 11.7)	15.8 (12.5, NR)	0.084
*NPM1*	10.3 (7.2, 14.3)	7.3 (4.5, NR)	0.260	9.6 (5.1, 14.2)	6.4 (3.8, NR)	0.698
*ASXL1*	8.9 (6.9, 13.2)	18.7 (4.8, NR)	0.229	7.1 (5.1, 14.1)	14.6 (10.0, NR)	0.498
*STAG2*	8.8 (5.8, 13.2)	19.5 (11.9, NR)	0.469	8.6 (5.6, 14.2)	11.1 (5.1, NR)	0.395
Any DNA methylation gene^b^	8.8 (5.4, 18.7)	11.1 (5.8, 15.3)	0.357	6.7 (4.9, 14.2)	12.5 (4.3, 17.6)	0.299

^a^*FLT3*-ITD and *FLT3-*TKD

^b^Includes *IDH1, IDH2, DNMT3A, TET1*, and *TET2*

*MUT* mutant gene(s), *NR* not reached, *OS* overall survival, *WT* wild-type gene(s)

Survival comparisons between the azacitidine and CCR arms indicated that patients with mutant *TP53* or *NRAS* treated with azacitidine had nominally better median OS than their counterparts in the CCR arm: median OS was 7.2 vs 2.4 months, respectively, for patients with mutant *TP53*, and 11.8 vs 4.3 months for those with mutant *NRAS* (Table 2). Conversely, azacitidine-treated patients with *TET2* mutations had worse OS outcomes than CCR-treated patients with the mutation (median OS 9.6 vs 11.1 months, respectively; *P* = 0.036). Median OS was similar between treatment groups for patients with a mutation in any of the DNA methylation genes.

**Table 2:** Median OS between treatment arms (azacitidine vs CCR) for the most frequently mutated genes and genes involved in DNA methylation

Mutated gene	AZA	CCR	Stratified *P* Value
	Median OS, months (95%CI)	Median OS, months (95%CI)	
*TP53*	7.2 (3.9, 18.6)	2.4 (1.5, 7.1)	0.093
*NRAS*	11.8 (7.7, NR)	4.3 (2.3, NR)	0.151
*FLT3* ^a^	5.4 (4.5, NR)	6.4 (3.8, NR)	0.271
*TET2*	9.6 (4.5, 13.5)	11.1 (2.8, NR)	0.036
*IDH2*	12.6 (4.4, NR)	12.5 (5.6, NR)	0.429
*DNMT3A*	12.6 (7.0, 20.8)	10.3 (3.8, NR)	0.624
*RUNX1*	13.5 (8.8, NR)	6.4 (3.8, NR)	0.496
*NPM1*	7.3 (4.5, NR)	12.5 (4.3, 17.6)	0.726
*ASXL1*	18.7 (4.8, NR)	14.6 (10.0, NR)	0.643
*STAG2*	19.5 (11.9, NR)	11.1 (5.1, NR)	0.722
Any DNA methylation gene^b^	11.1 (5.8, 15.4)	12.5 (4.3,17.6)	0.248

^a^*FLT3*-ITD and *FLT3-*TKD

^b^Includes *IDH1, IDH2, DNMT3A, TET1*, and *TET2*

*NR* not reached

The influence of VAFs of mutant *TP53*, *NRAS*, *FLT3*-TKD, and *TET2* at baseline on OS *vs*. wild-type genes in the Cox model stratified by treatment showed a significant increase in relative hazards on OS with increasing mutant *TP53* (*P* < 0.0001) and *TET2* (*P* = 0.042) VAFs (Supplementary Figure 3). In individual treatment arms, the influence of increased *TP53* VAF on OS vs wild-type was both negative and significant in the azacitidine and CCR arms but was much stronger in the CCR arm (*P* < 0.0001 vs *P* = 0.058 in the CCR vs azacitidine arms, respectively) with higher relative hazard on OS in the CCR arm at comparable VAF levels. There was a significant correlation between increased mutant *TET2* VAF and OS hazard in the azacitidine arm (*P* = 0.0091) but no VAF influence in the CCR arm (*P* = 0.97) (Supplementary Figure 4).

## Discussion

Prognosis is dismal for older patients with AML and Adverse-risk cytogenetics, including those with complex or monosomal karyotypes. Approximately one-third of patients in AZA-AML-001 had an Adverse karyotype; median OS among azacitidine-treated patients was almost double that of patients treated with CCR. Similarly, azacitidine-treated patients with monosomal or complex karyotypes had 35 and 36% reduced risks of death, respectively, compared with similar patients who received CCR.

Deletions of part or all of chromosomes 5, 7, or 17 occur in 5–10% of all patients with AML, are often associated with complex and monosomal karyotypes, and carry a poor prognosis [11–13]. They are more common in older patients and were the most frequent cytogenetic abnormalities in the AZA-AML-001 study population, occurring in 10–19% of patients. These chromosomal defects are frequently associated with multilineage dysplasia in bone marrow, poor response to chemotherapy, and high relapse rate [14]. In the current analyses, median OS was approximately doubled in azacitidine-treated patients with chromosome 5, 7, or 17 abnormalities compared with similar patients who received CCR. Similar to reporting in MDS [15], patients with AML with chromosome 7 abnormalities fared particularly well with azacitidine, with a median OS improvement of 4.4 (± 0.3) months compared with CCR. Analogous effects with azacitidine for treatment of AML and MDS would not be unexpected, as chromosome 5, 7, and 17 defects are diagnostic features of AML with myelodysplasia-related changes (AML-MRC) [4, 16]. The majority of all patients in AZA-AML-001 (54%) were identified as having AML-MRC upon central cytogenetic review [14]. Better outcomes with azacitidine in patients with these specific cytogenetic abnormalities in the current analysis are consistent with improved survival reported for all azacitidine-treated patients with AML-MRC in this study, who showed a median OS prolonged by 4.0 months compared with CCR (8.9 vs 4.9 months; HR 0.74 [95%CI 0.57, 0.97]) [14].

The genomic landscape differs between younger and older patients with AML [2]. Mutational frequencies in the AZA-AML-001 “biomarker” population were as might be expected for older patients [17–20]. Mutations in genes encoding epigenetic modifiers, such as *DNMT3A*, *TET2*, and *IDH2*, are more common in older patients and are usually acquired early in the evolution of the disease, often present in the founding clone. Similarly, mutations that are acquired later (e.g., *NPM1* and *FLT3*) occurred less frequently than what has been reported for other large AML patient cohorts that included younger patients [3, 21].

In this analysis, mutations in four genes were shown to significantly impact survival within treatment arms: mutant *TP53* and *NRAS* in the CCR arm, and mutant *FLT3* and *TET2* in the azacitidine arm. Within the CCR arm, *TP53* and *NRAS* mutations were associated with significantly reduced OS compared with patients with wild-type genes. *TP53* mutations, which occur in ~5–8% of all patients with AML [22, 23], are more frequently observed in older patients (21% of patients in the current study) and patients with abnormalities of chromosomes 5, 7, or 17p, are associated with complex karyotype, and generally indicate a poor prognosis in hematologic malignancies regardless of treatment choice [3, 22, 24–29]. However, in keeping with the current study, it has been suggested that hypomethylating agents (HMAs) may be more effective than conventional care in patients with these mutations. In a study of decitabine treatment in patients with AML or MDS, those with *TP53* mutations had a 100% response rate compared with a 41% response rate in patients with wild-type *TP53* [30]. During decitabine treatment, *TP53* VAF decreased rapidly to <5% (though the mutation was never completely cleared); this was accompanied by bone marrow blast clearance in many instances. In the current analysis, median OS was prolonged by almost 5 months in patients with pretreatment *TP53* mutations who received azacitidine compared with similar patients who received CCR. There was a significant correlation between higher *TP53* VAF at baseline and decreased survival compared with patients with wild-type *TP53* in both treatment arms, but the relative hazard was much greater in the CCR arm. The prognostic effects of *NRAS* mutations, which occur in ~15% of patients with AML (12% of patients in this study), typically at hotspot regions at codons 12, 13, and 61, are less clear. Their clinical implications may depend on the co-mutational context in which they occur, and patterns of *NRAS* co-mutations can vary by hotspots within genes [23, 31, 32]. For example, a recent study showed *NPM1* mutations to be preferentially associated with *NRAS*^G12/13^ but not with *NRAS*^Q61^ and that OS outcomes were more favorable when *NRAS*^G12/13^ mutations were accompanied by *NPM1* and *DNMT3A* mutations [3].

*TET2* mutations occur in about 7–25% of patients with AML (25% in this older patient population) [27, 33]. Although median OS within the azacitidine arm for patients with *TET2* mutations differed by only 0.1 month compared with those without the mutation, the Kaplan–Meier curve separated after the estimated median, leading to a statistically significant difference in OS between the two groups. Median OS was ~1.5 months longer in CCR-treated patients with *TET2* mutations than in similar azacitidine-treated patients, which was unexpected based on a pathological feature associated with *TET2* mutations (hypermethylation of DNA) and the purported activity of azacitidine (DNA demethylation) [34–36]. However, as there were a relatively small number of azacitidine-treated patients with *TET2* mutations in this analysis (*n* = 22) this finding requires further confirmation in a larger patient population. When taken as a group, mutations in genes that regulate DNA methylation did not influence median OS with azacitidine or CCR treatment. The prognostic consequences of mutations in *FLT3* may vary based on co-occurring mutations; for example, when present with an *NPM1* mutation in younger patients, prognosis is somewhat better than if accompanied by wild-type *NPM1* [37]. Approximately 20% of AML patients present with *FLT3* mutations although they are more common in younger patients with normal karyotype (only 12% of patients in the current study had an *FLT3* mutation) [23, 38]. There was no statistical difference between azacitidine and CCR treatment in median OS of patients with *FLT3* mutations, but within the azacitidine arm, presence of a *FLT3* mutation at baseline was associated with poorer OS compared with wild-type *FLT3*.

Class-defining *NPM1* and a provisional entity, *RUNX1* [4], were among the most commonly mutated genes in the biomarker cohort. Although differences in survival were not statistically significant within treatment arms compared with the wild-type genes, mutations in *NPM1* appeared to confer somewhat poorer survival, in contrast to what has been shown in other AML cohorts [39, 40], in both the azacitidine and CCR treatment arms. Moreover, mutations in *RUNX1* were associated with slightly better median OS in this analysis, although they have been associated with poorer prognosis in other studies [41, 42]. The number of patients in this analysis with mutant *NPM1* (*n* = 25) or *RUNX1* (*n* = 28) were relatively small, and these outcomes highlight a potential limitation of the data; namely, effects of isolated mutations or chromosomal defects provide only limited information by not considering cooperating pathogenic mechanisms at work in any given patient. Another limitation of this analysis is that changes in molecular and cytogenetic abnormalities during treatment were not captured.

The extraordinary heterogeneity and complexity of pathogenic mechanisms found in AML and the interplay among them in individual patients have made finding a cure–or even effective treatment–challenging. Nevertheless, increasing understanding of the genomic basis of AML and the introduction of new targeted therapies may allow the use of rational combination treatment regimens that include broadly effective agents such as azacitidine and an agent targeting a specific pathogenic pathway to improve patient outcomes. Studies in AML of azacitidine in combination with the BCL2 inhibitor, venetoclax (ClinicalTrials.gov NCT03466294), the mutant IDH inhibitors, enasidenib and ivosidenib (NCT02677922), and the mutant *FLT3* inhibitors, gilteritinib (NCT02752035) and quizartinib (NCT01892371), are currently ongoing.

The data presented here suggest that azacitidine may be the preferred treatment for older patients with newly diagnosed AML with Adverse-risk cytogenetics who are not candidates for intensive chemotherapy, particularly those with chromosome 5, 7, and/or 17 abnormalities, and with complex or monosomal karyotypes. Moreover, older AML patients with *TP53* or *NRAS* mutations may have prolonged survival when treated with azacitidine rather than with CCR. Outcomes of studies evaluating azacitidine as the backbone of combination regimens with targeted treatments are eagerly anticipated.

## Electronic supplementary material

Supplemental File
