# Supplementary material for: Cytogenetics and gene mutations influence survival in older patients with acute myeloid leukemia treated with azacitidine or conventional care
Source: Leukemia. 2018 Oct 1;32(12):2546–57. doi: 10.1038/s41375-018-0257-z (PMC6286388; doi:10.1038/s41375-018-0257-z)
Supplement: Supplementary file 1 — Supplemental File [file 41375_2018_257_MOESM1_ESM.docx]

**Supplementary Table 1. Baseline demographic and disease characteristics of patients in the “Biomarker Cohort” with available baseline mutational data**

| **Characteristic** | **AZA**  **(n=83)** | **CCR**  **(n=73)** |
| --- | --- | --- |
| **Age**, median (range) | 76.0 (64-89) | 75.0 (65-88) |
| Age ≥ 75, n (%) | 48 (58) | 38 (52) |
| **Gender**, n (%) |  |  |
| Male | 44 (53) | 44 (60) |
| Female | 39 (47) | 29 (40) |
| **Prior diagnosis of MDS**, n (%) | 19 (23) | 10 (14) |
| **WHO 2008 AML classification**, n (%) |  |  |
| AML not otherwise specified | 52 (63) | 37 (51) |
| AML with myelodysplasia-related changes | 27 (33) | 29 (40) |
| Therapy-related myeloid neoplasms | 2 (2) | 5 (7) |
| AML with recurrent genetic abnormalities | 2 (2) | 2 (3) |
| **BM blasts (%)**, median (range) | 73.0 (3-100) | 71.0 (8-100) |
| **ECOG PS**, n (%) |  |  |
| Grade 0-1 | 66 (80) | 57 (78) |
| Grade 2 | 17 (20) | 16 (22) |
| **2010 ELN cytogenetic risk**, n (%) |  |  |
| Intermediate I/II | 56 (67) | 41 (56) |
| Adverse | 27 (33) | 32 (44) |
| **WBC (10^9^/L)**, median (range) | 3.2 (0.6-19.5) | 2.3 (0.4-22.6) |
| **ANC (x10^9^/L)**, median (range) | 0.3 (0.0-4.9) | 0.2 (0.0-4.5) |
| **Hemoglobin (10^9^/L)**, median (range) | 9.6 (5.0-13.4) | 9.4 (7.3-14.4) |
| **Platelets (10^9^/L)**, median (range) | 54.0 (7-585) | 73.0 (9-327) |
| ANC, absolute neutrophil count; AZA, azacitidine; BM, bone marrow; CCR, conventional care regimens; ECOG PS, Eastern Cooperative Oncology Group performance status; ELN, European LeukemiaNet; MDS, myelodysplastic syndromes; WBC, white blood cells count; WHO, World Health Organization | | |

**Supplementary Figure 1. Significantly co-mutated gene pairs and mutually exclusive gene pairs**

| **Gene 1** | **Gene 2** | Gene 1^wt^: Gene 2^wt^ | Gene 1^mut^: Gene 2^wt^ | Gene 1^wt^:  Gene 2^mut^ | Gene 1^mut^: Gene 2^mut^ | Co-mutation *P* value | Mutually exclusive  *P* value |
| --- | --- | --- | --- | --- | --- | --- | --- |
| *EZH2* | *ASXL1* | 134 | 5 | 14 | 3 | 0.043 | 0.995 |
| *GNAS* | *ETV6* | 147 | 5 | 2 | 2 | 0.010 | 1.000 |
| *IDH2* | *IDH1* | 106 | 36 | 14 | 0 | 1.000 | 0.021 |
| *NPM1* | *ASXL1* | 114 | 25 | 17 | 0 | 1.000 | 0.043 |
| *NPM1* | *FLT3* | 122 | 16 | 9 | 9 | 0.000 | 1.000 |
| *NPM1* | *KIT* | 131 | 23 | 0 | 2 | 0.025 | 1.000 |
| *NRAS* | *GATA2* | 132 | 14 | 6 | 4 | 0.017 | 0.998 |
| *PHF6* | *ASXL1* | 138 | 1 | 15 | 2 | 0.032 | 0.999 |
| *PTPN11* | *NPM1* | 123 | 8 | 20 | 5 | 0.037 | 0.993 |
| *PTPN11* | *NRAS* | 129 | 9 | 14 | 4 | 0.046 | 0.992 |
| *RAD21* | *KIT* | 152 | 2 | 1 | 1 | 0.038 | 1.000 |
| *RUNX1* | *NPM1* | 103 | 28 | 25 | 0 | 1.000 | 0.004 |
| *SF3B1* | *RUNX1* | 123 | 5 | 23 | 5 | 0.017 | 0.997 |
| *STAG2* | *CEBPA* | 140 | 13 | 0 | 3 | 0.001 | 1.000 |
| *STAG2* | *RUNX1* | 119 | 9 | 21 | 7 | 0.010 | 0.998 |
| *TET2* | *IDH1* | 103 | 39 | 14 | 0 | 1.000 | 0.014 |
| *TET2* | *IDH2* | 85 | 35 | 32 | 4 | 0.995 | 0.020 |
| *TP53* | *ASXL1* | 107 | 32 | 17 | 0 | 1.000 | 0.016 |
| *TP53* | *DNMT3A* | 86 | 28 | 38 | 4 | 0.992 | 0.028 |
| *TP53* | *IDH1* | 110 | 32 | 14 | 0 | 1.000 | 0.034 |
| *TP53* | *IDH2* | 91 | 29 | 33 | 3 | 0.993 | 0.028 |
| *TP53* | *MLL* | 123 | 28 | 1 | 4 | 0.006 | 1.000 |
| *TP53* | *NPM1* | 99 | 32 | 25 | 0 | 1.000 | 0.002 |
| *TP53* | *RUNX1* | 96 | 32 | 28 | 0 | 1.000 | 0.001 |
| *U2AF1* | *ETV6* | 142 | 10 | 1 | 3 | 0.002 | 1.000 |
| *U2AF1* | *IDH2* | 107 | 13 | 36 | 0 | 1.000 | 0.028 |

**
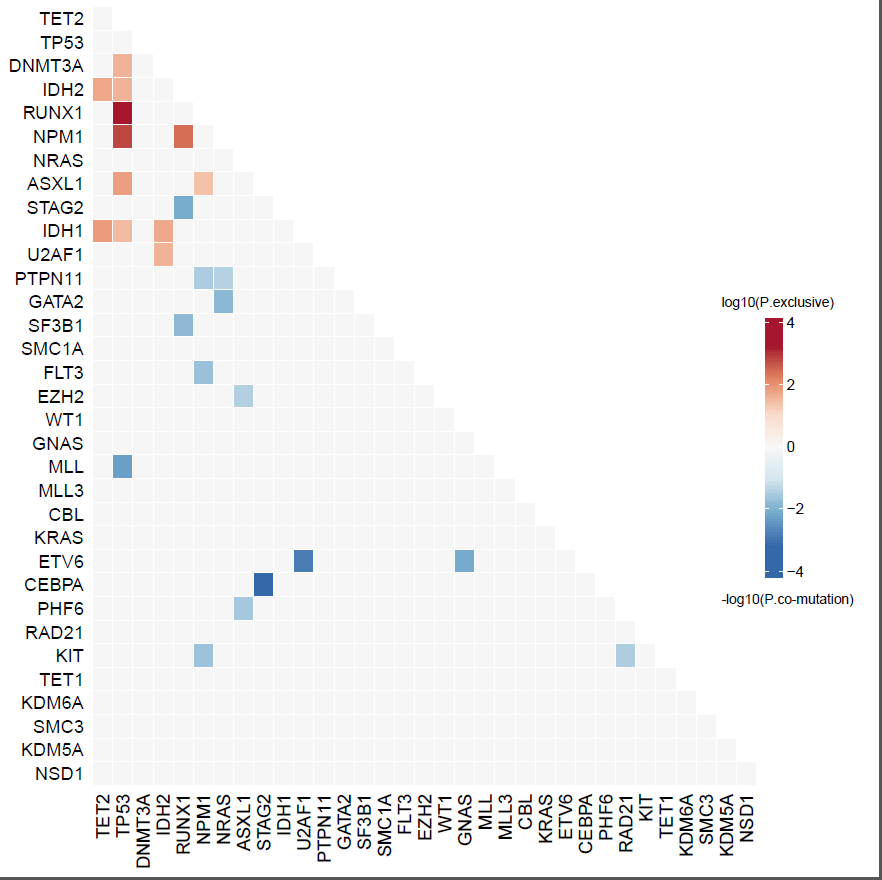
**

*P* values from Fisher’s exact test

**Supplementary Figure 2. Gene mutations not significantly correlated with overall survival within treatment arms (mutant *vs.*** **wild-type)**

**Supplementary Figure 3. Relative hazards on overall survival with increasing variant allele frequencies of relevant mutant genes compared with survival of patients with wild-type genes.** *FLT3* not shown; *FLT3*-ITD analyzed by capillary electrophoresis PCR precluding assessmenet of variant allele frequency. Treatments (azacitidine, CCR) were stratification factors in the Cox model.


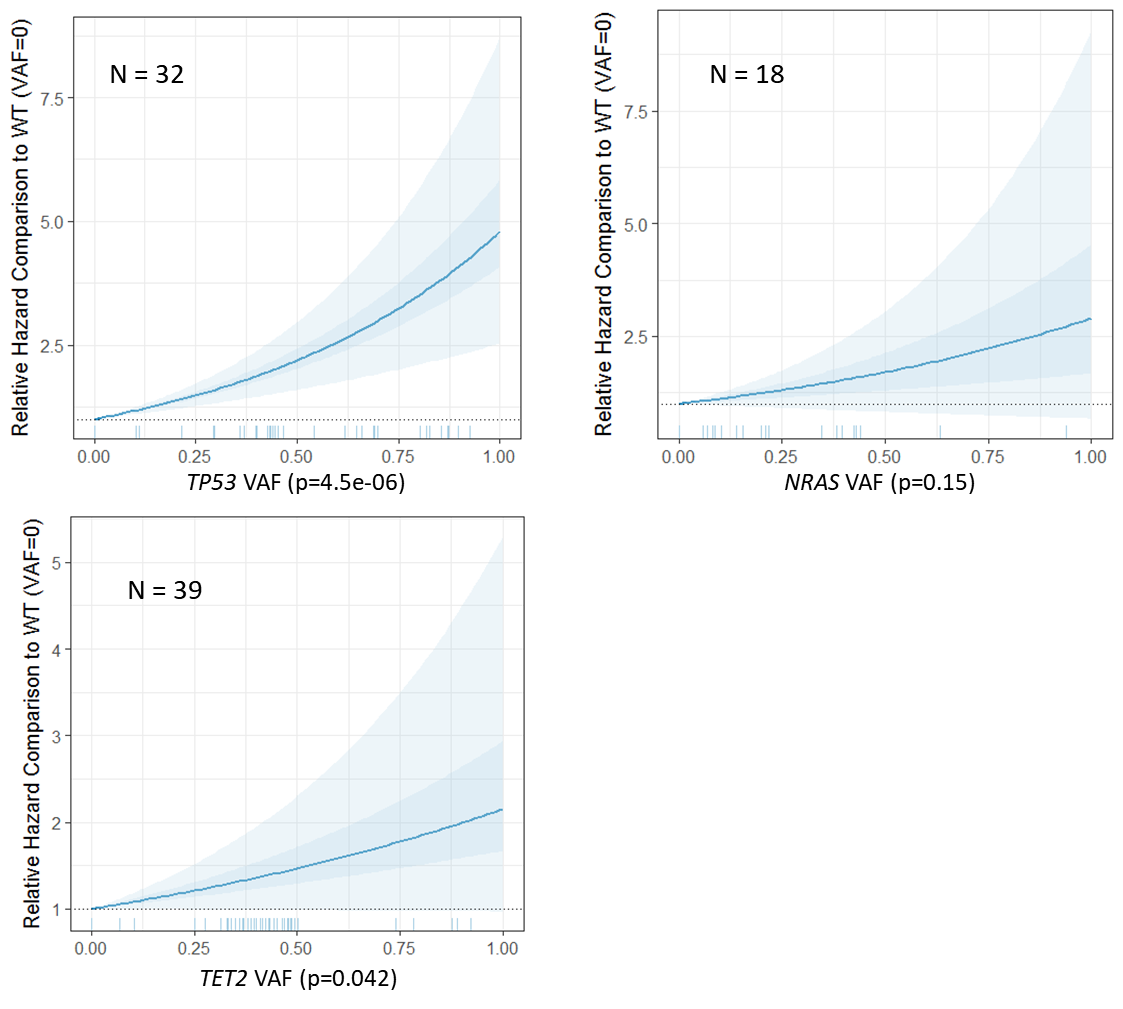


**Supplementary Figure 4. Relative hazards on overall survival with increasing variant allele frequencies of relevant mutant genes compared with survival of patients with wild-type genes by treatment arm.** *FLT3* not shown; *FLT3*-ITD analyzed by capillary electrophoresis PCR precluding assessmenet of variant allele frequency.

**
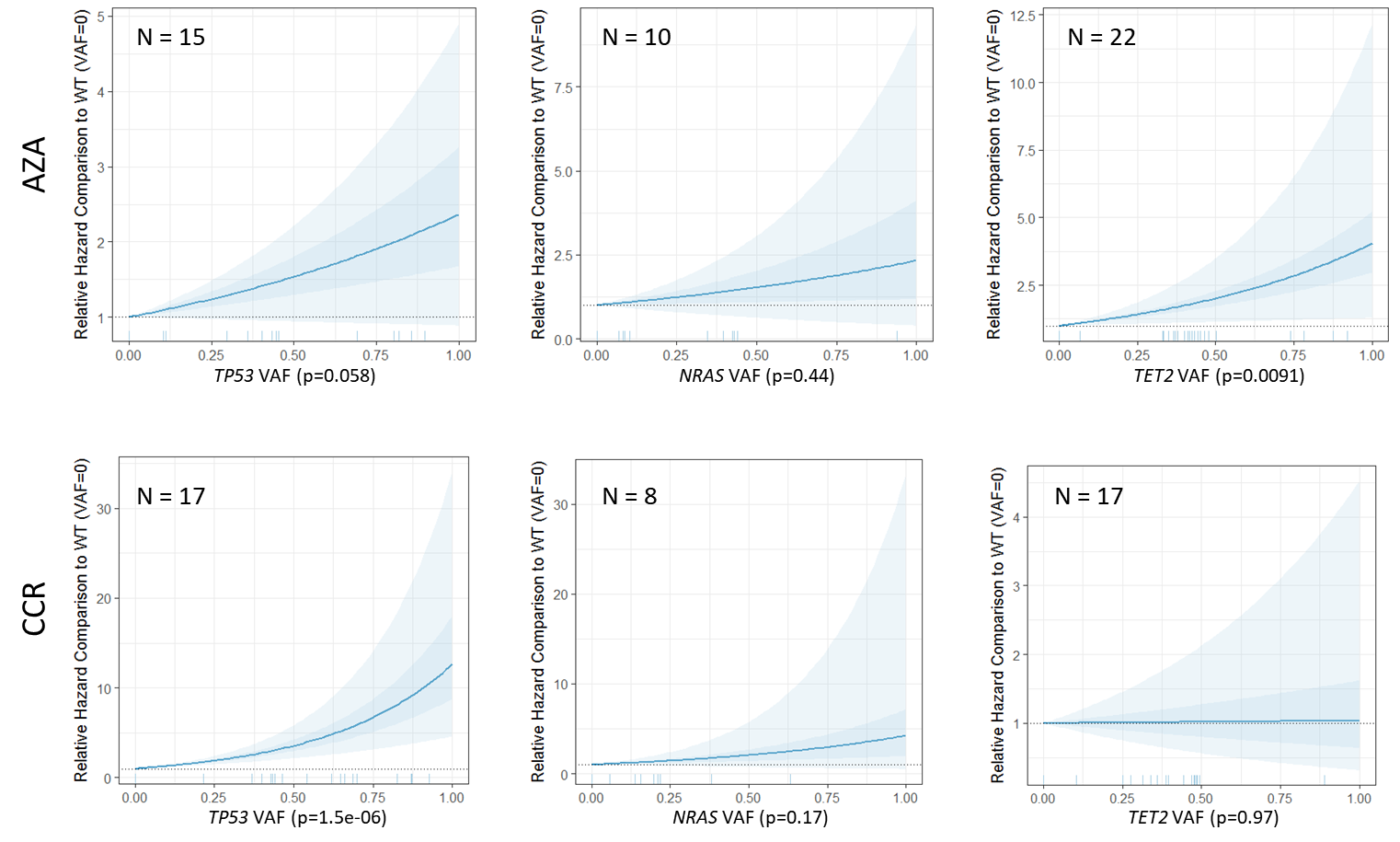
**
